# Leprosy treatment dropout: a sistematic review

**DOI:** 10.1186/1755-7682-6-34

**Published:** 2013-08-30

**Authors:** Régio José Santiago Girão, Nara Lívia Rezende Soares, Juliana Viana Pinheiro, Giuliano da Paz Oliveira, Sionara Melo Figueiredo de Carvalho, Luiz Carlos de Abreu, Vitor E Valenti, Fernando Luiz Affonso Fonseca

**Affiliations:** 1 Faculdade de Medicina do ABC, Santo André, SP, Brazil; 2 Faculdade de Saúde, Ciências Humanas e Tecnológicas do Piauí, Teresina, PI, Brazil; 3 Universidade Federal do Ceará, Barbalha, CE, Brazil; 4 Universidade Federal de São Paulo, São Paulo, SP, Brazil; 5 Universidade Estadual Paulista – UNESP, Marília, SP, Brazil

**Keywords:** Leprosy, Patient dropout, Treatment, Adhesion

## Abstract

**Background:**

Leprosy is a chronic infectious disease endemic in some undeveloped areas, and still represents a public health problem in Brazil. Therefore, the control of this endemic disease depends necessarily on the institution of correct treatment and containment of treatment dropout. This study aims to conduct a systematic review of published studies on treatment dropout of leprosy.

**Methods:**

We conducted a systematic review of articles on treatment dropout of leprosy, published between january 2005 and april 2013, on MEDLINE and SciELO databases. The search was performed using the MeSH terms: “leprosy”; “patients dropouts” and the keywords: “leprosy, treatment” and “noncompliance, leprosy” in association, beside the equivalents in Portuguese.

**Results:**

There were originally 196 references. After analyzing the titles and abstracts of articles, 20 articles were obtained and included in the final sample.

**Discussion:**

Leprosy is a notifiable disease known as its disfiguring capability and the high rate of non-compliance to treatment. The low adhesion is responsible for the remaining potential sources of infection, irreversible complications, incomplete cure and, additionally, may lead to resistance to multiple drugs. Many factors are responsible for the interruption or dropout treatment: socioeconomic factors, education level, knowledge about the disease, lack of efficiency of health services, demographics, side effects of drugs, alcoholism, among others.

**Conclusion:**

The recent scientific literature about the subject diverge regarding the factors that most affect the dropout problem in treating leprosy patients. However, better integration between professionals and users, and greater commitment of the patient, are common points among the authors of the studies.

## Abstract (pt)

**Resumo:**

**Introdução**

A hanseníase é uma doença infecciosa crônica endêmica em algumas áreas subdesenvolvidas, e ainda representa um problema de saúde pública no Brasil. Portanto, o controle da endemia depende necessariamente da instituição do tratamento correto e contenção do abandono do tratamento. Este estudo tem como objetivo realizar uma revisão sistemática dos estudos publicados sobre abandono do tratamento da hanseníase.

**Métodos**

Foi realizada uma revisão sistemática de artigos sobre o abandono do tratamento da hanseníase, publicados entre janeiro de 2005 e abril de 2013, nas bases de dados MEDLINE e SciELO. A pesquisa foi realizada utilizando os termos MeSH: “leprosy”; “patients dropouts” e as keywords: “leprosy, treatment” e “noncompliance, leprosy”, em associação, além das equivalentes em Português.

**Resultados**

Foram originalmente 196 referências. Após a análise dos títulos e resumos de artigos, 20 artigos foram obtidos e incluídos na amostra final.

**Discussão**

A hanseníase é uma doença de notificação obrigatória conhecida pelo seu alto potencial de levar a incapacidades, e está associada a uma elevada taxa de não-adesão ao tratamento. A baixa aderência é responsável pelos focos de infecção residuais, aumento de danos irreversíveis, cura incompleta e pode levar a resistência a múltiplas drogas. Muitos fatores são responsáveis pela interrupção ou o abandono do tratamento: fatores socioeconômicos, escolaridade, conhecimento sobre a doença, a falta de eficiência dos serviços de saúde, demografia, os efeitos colaterais das drogas, alcoolismo, entre outros.

**Conclusão**

A literatura científica recente sobre o assunto diverge sobre os fatores mais associados ao abandono do tratamento de pacientes com hanseníase. No entanto, uma melhor integração entre os profissionais e usuários e maior comprometimento do paciente, são pontos comuns entre os autores dos estudos.

## Introduction

Leprosy (also known as Hansen’s disease) is a chronic infectious disease with particular epidemiological and clinical characteristics. It is caused by the bacillus *Mycobacterium leprae*, a intracytoplasmic parasite, affecting mainly the skin and/or peripheral nerves [1]. The primary source of human leprosy is the transmission by untreated patients. The main location for the spread of infection is in the family setting where contact is constant. In eventual contact with untreated patients, only 2-5% will become ill [2].

Leprosy is considered a public health problem in Brazil and worldwide. In 2010, 211,903 cases of leprosy were notified by World Health Organization (WHO) in 141 countries or territoriums [3]. In 1991, WHO adopted a resolution to eradicate leprosy as a public health problem, for that would be required a prevalence of less than 1 per 10,000 people. However, due to failures in policies to combat the endemic, Brazil has shown in 2012, according to the Brazilian Public Department of Health the second highest prevalence in the world (18,22 per 100,000 people), following only India [4].

Based on epidemiological aspects it is known that leprosy represents a public health problem in Brazil. Despite the undeniable advances of public actions to control Hansen’s disease, it stills an important problem to be faced in Brazil and worldwide, demanding more realistic goals and strategies for their control. The control of this endemic disease needs necessarily of correct treatment and containment of treatment dropout [4].

To that end, the Brazilian Public Department of Health recommends epidemiological and operational indicators for adequate monitoring of leprosy control programs, including the treatment abandonment that was included in Primary Care Pact between the federal government, states and municipalities. According to its definition, dropout is the situation when the patient did not complete the number of doses on time and when did not attend the service in the last 12 months [4].

The issue of adherence to leprosy’s treatment is closely linked to disease control. Identifying factors associated with abandonment is important to define high risk groups that should be followed more carefully during multidrug therapy (MDT). These actions not only help in reducing the dropout rate but also mitigating the risk of developing resistance to MDT [5].

Different studies have been developed by approaching the factors of non-adherence to treatment and neglect of patients with chronic diseases, but there are few studies that discuss the problem of non-adherence among patients with leprosy. In this regard, this study aims to conduct a systematic review of published studies on treatment dropout of leprosy. A study of this magnitude deepens the knowledge about this theme and becomes a source of information for the promotion of public policies measures to combat therapy abandonment and consequently control the disease.

## Methodology

A qualitative systematic review of scientific literature was made, regarding the leprosy treatment dropout.

The search for articles was performed on PubMed and SciELO databases in February 2013 and was limited to articles published between 01/01/2005 and 04/01/2013. The initial strategy was based on the intersection of the following terms.

Initially, the search terms browsed in Pubmed database were:

#1 “Leprosy” (MeSH term);

#2 “Patients dropouts” (MeSH term);

#3 “leprosy,treatment” (keyword)

#4 “noncompliance, leprosy”(keyword)

The following searches were performed: #1 AND #2, #3, #4. The articles found by using this strategy were reviewed on two separate occasions in order to ensure the adequacy of the sample.

Similar search strategy was performed in SciELO database, using the terms mentioned above and their equivalents terms in Portuguese.

The analysis of articles followed the inclusion and exclusion criteria previously established. We included: a) articles that presented on the abstract at least a combination of the terms established b) manuscripts in English or Portuguese, c) studies on treatment dropout of leprosy d) original articles, e) prospective or retrospective observational studies (analytical or descriptive, except case reports), experimental or quasi-experimental. Exclusion criteria were: a) other study designs, such as case reports, case series, literature reviews, b) non-original studies including editorials, book reviews and letters to the editor.

Each paper of the sample was read in its entirety, and data relevant to this research were extracted and included a spreadsheet containing authors, year of publication, sample description and main findings of the study. In order to provide a better analysis, articles were grouped in 2 themes: multidrug treatment and factors for the treatment abandonment.

## Results

**Figure 1:**
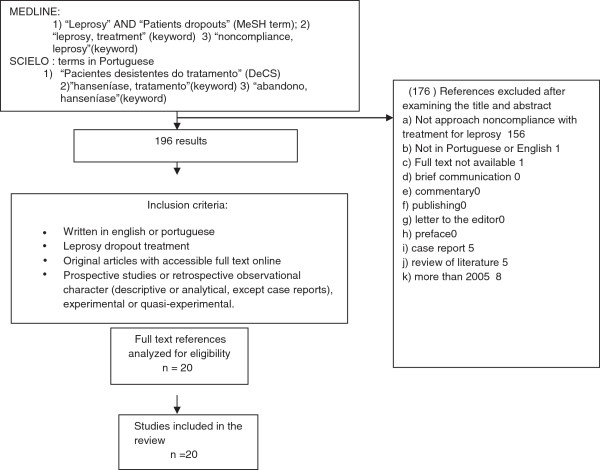
Flow chart summarizing the procedure for selection of studies for review.

Initially, the search of databases as registered above resulted in 196 papers. After analyzing the titles and abstracts of the papers, according to the eligibility criteria, 176 papers were excluded and 20 were chosen and included in the final sample (Figure 1). The articles in the MEDLINE database and SCIELO met the inclusion criteria established for this review. In the sequence, Table 1 presents an overview of all the studies included in the final sample and all data collected and used during sample analysis.

**Table 1:** Presents an overview of all the studies included in the final sample and all data collected and used during sample analysis

**Author(year)**	**Journal**	**Sample**	**Main finding**
PSS Rao [15]	Indian journal of leprosy	6.291 patients with leprosy	Adherence to MDT and its successful completion are equally important. Unfortunately, due to a number of personal, psychosocial, economic, medical and health service factors, a significant number of patients become irregular and default from MDT. In this paper, the extent of such defaulting was based on a study of six leprosy mission hospitals. Nearly 50% of patients closer to the hospitals as compared to 60% beyond have defaulted. Patients from outside the district had significantly higher default rate for all types of leprosy cases as compared to patients living close to the centres. Motivation, counselling and frequent contact with the patients will help.
Heukelbach et al. [13]	PLoS Neglected Tropical Diseases	1.635 leprosy patients of 78 cities.	Was performed a population-based study in municipalities in Tocantins State, Brazil, Was used two outcomes for assessment of risk factors: defaulting (not presenting to health care center for supervised treatment for >12 months); and interruption of MDT. In total, 3.0% patients defaulted, and 18.2% interrupted MDT. Defaulting and interruption of MDT are associated with some poverty-related variables such as family income, household size, and migration. Intermittent problems of drug supply need to be resolved, mainly on the municipality level. MDT producers should consider oral drug formulations that may be more easily accepted by patients. Thus, an integrated approach is needed for further improving control, focusing on vulnerable population groups and the local health system. This study showed higher rates of discontinuation treatment in young males (34.4%), when the data were stratified by sex.
Trindade et al. [18]	Caderno de Saúde Coletiva do Rio de Janeiro	56 patient who dropout leprosy treatment	Shows that alcoholism is associated significantly with treatment dropout of leprosy in Paraíba, Brazil. The study shows that 53.4% of patients who dropout treatment made regular use of alcohol. Among those who took treatment regularly, none made regular use of alcohol. Those patients who abandoned treatment had lower income. The average income of patients who abandoned the treatment was R$ 300.00 monthly, while those who continued the treatment dimension regular income was R$ 700.00.
Honrado et al. [12]	Dermatology Clinic	233 leprosy patients	The rate of noncompliance among those who have not had their drugs from health centers (57.1%) was significantly higher than among those who obtained their drugs from health centers (27.3%). Even so the rate of treatment abandonment in patients that it was not told the name of the disease was significantly higher than those previously reported (44.4% versus 23.6%, respectively).
Kar S. et al. [10]	Journal of Neurosciences in Rural Practice	254 leprosy cases	The distribution of defaulters to treatment in relation to the state of alphabetization, per capita income and socioeconomic status in India as compared to the control group reflects that the majority (32.28%) had attended school leaving certification exam (completed primary education), per capita income between R 500–749 (30.71%) and belonging to social class IV (33.86%) and V (30.71%).
Luna et al. [22]	Revista Brasileira de Enfermagem	6 patiens in leprosy treatment.	In research conducted by Activity Life Model of Roper, Logan and Tierney identified the weakness in credibility by the patient regarding medical diagnosis and non-acceptance of the use of MDT as a tool to obtain the cure of the disease. Using the Activity Life Model with a focus on home visit was important because it provided a holistic approach to research, offered for leprosy patients, allowing a detailed knowledge of the issues involved in maintenance practices and healthy lifestyles. This study is important to help health services to meet the life activities of leprosy patients, and thus direct the actions related to these patients and contributing to the planning educational activities that encourage the individual to self-care in order to develop a life healthy.
Souza et al. [6]	International Journal of Dermatology	Leprosy patients	The study listed the main adverse factors that disturb the patient and lead to dropping out of treatment: anemia, agranulocytosis, neuropathy, hepatotoxicity, hyperpigmentation. Reaction episodes may present abruptly and cause a range of severe symptoms.
Chalise [20]	Journal Of The Nepal Medical Association	436 leprosy patients who failed to complete the cycle of MDT.	Was noted in the study that about 86% of non-adherence cases of treatment of leprosy have a wrong concept about the disease. It was also observed that the majority (94.7%) cases of noncompliance have a strong belief that the disappearance of the skin lesion is the only meaning of “cure the disease”. These two factors create a negative outcome in relation to treatment due to lack of necessary information about the disease.
Araújo e Oliveira [23]	Hansenologia Internationalis	Study with 57 patients with leprosy in irregular treatment.	The majority of those that were in irregular treatment were men (71.9%), between the ages of 20 and 49 years (54.3%), a productive age. The women who dropout treatment were concentrated between 50 and 69 years old.
Penna et al. [5]	Revista da Sociedade Brasileira de Medicina Tropical	1124 leprosy patients	Leprosy reaction episodes were recorded in 328 pacients (29.2%). Was observed that type 2 leprosy reactions were common, especially in those with lepromatous form. The treatment is prolonged and sometimes difficult, requiring multiple medications with diverse side effects that can result in dropout the tratment and increased the rates of leprosy reaction.
Hacker et al. [24]	Ciênc. saúde coletiva	1353 leprosy patiens of Rio de Janeiro	The paper compares the epidemiological data on leprosy patients living in two municipalities with socioeconomic profile and level of endemicity different, Rio de Janeiro and Duque de Caxias, Brazil and were followed by the reference centers. There were no statistically significant differences regarding gender, disability level, reaction in the diagnosis, abandonment and regularity of treatment. The differences found between the patients monitored in a single center of reference, could be partly related to contextual differences between the municipalities.
Lustosa et al. [14]	Revista da Sociedade Brasileira de Medicina Tropical	107 patients	An observational study done in Piaui, Brazil, aiming to analyze the quality of life related to health of people in treatment of leprosy in Teresina - Piaui. A specific questionnaire for assessing quality of life that sought the determinants of decline in the quality of life for people with leprosy, outlining the profile sociodemographic, clinical and epidemiological patients interviewed. The study of the impact of leprosy on quality of life reinforces the need to implement more effective strategies for early diagnosis and to break the chain of disease transmission and to increase the commitment of the leprosy patient to his treatment.
Raposo et al. [19]	Revista da Sociedade Brasileira de Medicina Tropical.	Data from national basis.	The study evaluates epidemiological and operational program of leprosy in the period before and after the integration of primary care services in the city of Aracaju, Brazil. The proportion of cases with disability grade assessed at diagnosis, increased of 60.9% to 78.8% (p < 0.001), and the conclusion of the treatment, from 41.4% to 44.4% (p < 0.023); and a lower level of treatment default with a decrease from 5.64 to 3.35 (p < 0.008). Abandonment rates have decreased in the post-integration of primary care services.
Silva et al. [17]	Revista da Sociedade Brasileira de Medicina Tropical	42 patients with leprosy	This study was developed to evaluate the situation of leprosy in municipality of Buriticupu, State of Maranhão, Brazil. Was used the method of active search to identify new cases from 2008 to 2010. The study shows that the good therapeutic response is consequence of the effectiveness of MDT, the early diagnosis, the patient’s knowledge about the disease, the commitment of the patients to the therapy and trained health professionals.
Silva et al. [11]	Escola Anna Nery	14 healthcare Professional	This research discusses the experience of professionals basic health services in the municipality of Rio de Janeiro,Brazil, which perform activities of health education in Leprosy Control Program. This program aim to increase the knowledge of the patient about the disease and consequently increase the rates of treatment adhesion. The education activites also want to reduce the social prejudice.
Miranzi [16]	Revista da Sociedade Brasileira de Medicina Tropical	455 patients	This retrospective study describes the epidemiological profile of the population diagnosed with leprosy in the district of Uberaba, State of Minas Gerais, Brazil, from 2000 to 2006. It is estimated that only 1/3 of patients are notified and that among these, many are irregular treatment or drop out, increasing the impact of the disease.
Lira et al. [3]	The Brazilian Journal of Infectious Diseases	70 leprosy patients	It was observed that 57.1% of patients in the study had no difficulty adhering to treatment, while 38.6% reported little difficulty. This study shows that although patients who claim to be familiar with leprosy and its treatment, the Morisky-Green test clearly shows that they really had no knowledge of the principles of therapy, which is evidenced by the low level of adherence to treatment.
Ferreira et al. [8]	Revista Brasileira de Epidemiologia	Data from national basis.	Cross-sectional study of diagnosed cases of leprosy relapse in reference units from 2005 to 2007 in 5 municipalities in the state of Mato Grosso, Brazil.
			The diagnosis of recurrences occurred in early and late periods, compared to the time interval between initial treatment and recurrence rates correspond to the findings of other research. Several factors can influence the time to relapse: a clinical form, the therapeutic regimen, reactive episodes, irregular treatment and bacterial load.
Ferreira et al. [9]	Rev de Saúde Pública	159 pacientes	A retrospective case–control study in the state, of Mato Grosso-Brazil, to investigate the factors of recurrence that were related to living conditions, living habits, organization of health services, clinical and therapeutic regimens. Were associated with relapse of leprosy: living in rental housing; living in houses constructed of wood and mud; living with dwellings with more than five people; alcohol use disorder; irregular treatment; lack of knowledge about the disease/treatment; use of public transportation to get to the clinic; clinical form of the disease, and treatment regimen.
Weiand et al. [21]	Lepr Rev.	55 patients	The objective of this study was to measure medication adherence among outpatients attending an urban leprosy clinic in Hyderabad, India. Fifty two patients met the inclusion criteria for this study; 13 patients (25%) were non-adherent according to the Morisky scale questionnaire and 17 patients (33%) according to the urine spot test. 48% of patients were non-adherent on the basis of the urine spot test and/or the Morisky scale questionnaire.

## Discussion

### Multidrug therapy

Leprosy is a notifiable disease known for the potential physical disabilities. It is considered a very serious disease to public health, both in Brazil and in the world [3]. Initially, patients often have skin lesions and altered thermal sensitivity, sometimes progressing to altered sensitivity to pain and touch, muscle wasting, weakness in the limbs and, in rare cases, in advanced stages, painless burns or ulcers in hand or anesthetic foot [5].

Despite the old myths about the disease, its treatment is possible, especially if the diagnosis is correctly established in the early stages and when the patient undergo correctly the multidrug therapy recommended by the Brazilian Ministery of Health and standardized by WHO [5]. Multidrug therapy (MDT) for leprosy was introduced by WHO in 1982, in which paucibacillary patients are treated with rifampicin and dapsone for 6 months, while multibacillary patients receives rifampicin, dapsone and clofazimine for 12 months [5,6]. The introduction of multidrug therapy regimen in Brazil led to a reduction of up to 75% of leprosy’s prevalence rate. However, even with advances in treatment, leprosy is still a major public health problem in Brazil [7,8].

The success of the WHO strategy, vital for the elimination of leprosy (MDT strategy) depends largely on the efficiency of health services and patient commitment. The high rate of non-compliance to MDT has serious implications for leprosy control program, because it can contribute to development of drug resistance, and result in failure of the program [9-11].

Treatment dropout and a lack of multidrug therapy against leprosy are still major obstacles in the control the endemic diseases in many countries, with consequences for patients and control programs. The low adhesion is responsible for the remaining potential sources of infection, irreversible complications, incomplete cure and, additionally, may lead to resistance to multiple drugs. In Brazil, the number of patients who failed treatment was reduced from 3.148 patients in 2002 to 529 in 2009 (in these years, occurred about 49.000 and 37.500 new cases, respectively) [12-14].

### Factors for treatment dropout

Rao (2008) divided the causes of leprosy treatment abandonment in three categories: (a) personal (b) medical and (c) factors related to the healthcare centers. Personal factors include stigma and other social reasons, either psychological or economic, such as travel costs, loss of earnings, etc. Regarding medical problems were worsening illness, reactions, not disappearance of skin lesion or other symptoms, or even a feeling that patient was cured because the symptoms have disappeared [15].

For heuristic reasons, we divide the factors into: (I) personal factors: abording issues of quality of life, socio-economic and cultural needs of patients (II) medical factors: variables related to the treatment regimen and to the health service.

#### Personal factors

It is believed that understanding and patient behavior in relation to the adhesion to drug treatment are largely influenced by their socio-economic status and level of knowledge [16,17].

In a study by Kar and colleagues, the distribution of defaulters in relation to the state of alphabetization, per capita income and socioeconomic status in India as compared to the control group reflects that the majority (32.28%) had attended the school leaving exam certification (completed primary education) and per capita income between R 500–749 (30.71%) and belonging to social class IV (33.86%) and V (30.71%). Therefore, this study showed that there is a statistically significant association between the state of literacy, per capita income per month and socioeconomic status, with treatment result [10].

In a study performed in Paraíba, a Brazilian state, low income was not significantly associated with dropout due to the small sample. However, the average difference was amazing and it is believed that the small number of patients who abandonment treatment included in the sample have influenced this finding. This means that factors that define risk groups in general also apply to patients in the treatment of leprosy [18,19].

In the Philippines, was no found a link between socioeconomic factors and the probability of doupout treatment. Income was similar in patients who abandoned and those who completed treatment [12]. This difference may be related to the different social contexts in the Philippines, India and Brazil.

According to Honrado and collaborators, two factors have been shown to be associated with the probability of nonadherence: the a source of drugs and if the name of the disease was informed to the patient. It was revealed that the rate of noncompliance among those who have not had their drugs from health centers (57.1%) was significantly higher than those who obtained their drugs from health centers (27.3%). Those patients who obtained their supply of MDT from other sources than the health centers were 3.6 times more likely to become non-adherent than those whose medication was taken in the health centers [12].

This situation emphasizes the strategy of using such centers as the appropriate channel (if not ideal) for distribute the medicines of the MDT in the community. This action, together with the concept that most part of the treatment should be realized in the healthcare center controls the disponibility of drugs for MDT, which prevents accidental use, either for monotherapy or wrong combinations of medicines and also helps to prevent the development of multiple drug resistance. So, later, will help to ensure the success of the strategy for leprosy control [12].

Another factor analyzed by Honrado and colleagues was that the rate of noncompliance among those patients who did not know the name of the disease was significantly higher than those who were informed (44.4% versus 23.6%, respectively). Furthermore, the probability of abandoning the treatment of those who were not informed was 2.6 times higher than those who were told the name of his condition. This finding emphasizes the need that health professionals have to tell the patient the name of his affliction, in the hope that this strategy may improve adherence to treatment [12].

The study conducted by Trindade and colleagues, the number of users who said they had received enough information about the disease during treatment was slightly higher in the group that completed the regular treatment than the group that did not. This suggests that the abandonment is a result of the interactions of many factors which stands out health education [18].

A study by Chalise revealed that about 86% of cases of noncompliance, have no knowledge about the disease, approximately 39% are not sure what caused the disease, and only about 14% knew that the disease is caused by a bacterium or “microorganism”. It can also be observed that a significant proportion (33.3%) of dropout patients believed the disease was caused due to the desire of God or due to the sin committed by them in past lives. It was noted in the study that the community is still associating God’s desire and sin with the occurrence of the disease which can strongly contribute to the failure of the treatment of leprosy, as many believe that man and modern medicine can not challenge God [20]. It is interesting to note that the majority (94.7%) of cases of non-adherence to treatment has a strong belief that the disappearance of the sign / symptom is the only meaning of “cure the disease”. This also clearly indicates the low level of understanding of the treatment and therefore a negative result [20].

The understanding of leprosy patients about the disease is important to their quality of life [20]. Research on the performance of the drug indicated that if a patient understands his illness and its treatment as well, he is more likely to be motivated to take the entire course of treatment prescribed correctly [8,9]. The communication process consists of several angles and to types of communication be effected, verbal or non-verbal, it is necessary to place a meaning common to all participants. Through communication, the professional will inform the patient about the disease, treatment, and cure a variety of issues, if there is interest [21,22].

Available data about the influence of demographic variables on adherence to treatment are contradictory. In an analytical cross-sectional study conducted in Dhanusha, a district of Nepal, 183 leprosy patients were male (68.3% treated with MDT-MB) and 90 female (61.1% with MDT-MB). The study found that 79.2% of male patients completed treatment, while 34.4% of females not completed within the specified period. The study, however, showed significant associations between treatment completion status and gender [10].

However, the study by Heukelbach and colleagues, demographic data such as gender, age and civil status were not associated with poor adherence in this population. Interestingly, this study showed higher rates of discontinuation in young males (34.4%), when the data were stratified by sex. Men showed a difference of about two times compared to females of the same age (17.6%). This indicates that factors are multifaceted and that, in this case, young males should be considered as a vulnerable group regarding poor adherence, as men are already known to show a insufficient healthcare behavior [13]. The study by Araujo and Oliveira revealed that most male patients with irregular treatment were distributed in the productive age group (48.8% between 20 and 39 years) and this is one of the reasons that led to the non-attendance to scheduled service by the health centers [23]. This profile of the population with irregular situation and who abandoned the treatment demonstrates the high social cost of this situation on the population who have the greater productive capacity. It is necessary adequate treatment programs to the profile of the population as a way to avoid both treatment noncompliance as the abandonment itself.

The study by Trindade and colleagues found male predominance in leprosy dropout, however, due to the small number of cases, the difference was not statistically significant [18]. Those who noncompliance the treatment was predominantly unmarried, unlike what was found by Honrado (2008) study in which there was a predominance of married people in dropout patients [12].

In the study made in Paraíba, a Brazilian state, showed that alcoholism is associated with noncompliance to treatment, but showed no clinical variables associated to the occurrence of leprosy reactions, side effects of MDT or degree of disability. Alcohol consumption is traditionally recognized by population as prohibitive for medical treatment, what makes some people choose not to stop consuming it, rather than to therapy either by an unfavorable social and educational level (which makes recognition of importance of treatment) or by chemical dependency [18].

#### Medical factors

Studies performed in other parts of the world, have identified other risk factors for poor adherence. For example, in the study in the Philippines 40% of patients identified as the main cause for not completing the treatment regimen adverse effects experienced after drugs ingestion. These adverse effects were dizziness, headache, weakness, nausea, gastrointestinal discomfort, and others [12].

A study conducted by Souza et al. listed the main adverse factors that disturb the patient and lead to treatment dropout: anemia, agranulocytosis, neuropathy (associated with dapsone), hepatotoxicity (due to rifampicin and dapsone), hyperpigmentation (due to clofazimine). The reaction episodes may start abruptly and cause a wide range of intense symptoms such as inflammatory arthritis, erythema nodosum, fever, neuritis, paralysis of the limbs and even. The health professional must be prepared to promptly recognize and treat such complications, using tertiary services, if necessary [6].

Unlike the study conducted in the Philippines, another study in India showed that the most common cause for the default treatment of leprosy was the loss of working hours to go to the health center. This issue was mentioned by 33.1% of patients in the study, whereas adverse events were second with 26.0%. These refer patients who are delinquent due to lost working hours, when they come to receive drugs at the health center [10].

According to researchers from Nova Deli, on six missionary hospitals for leprosy, almost half of the patients who lived closest to the hospitals were dropouts, compared with patients who were beyond the hospitals, where 60% were delinquent. Patients from outside the district had significantly higher default rate in all types of leprosy cases, compared with patients who live close to the centers [15].

A study in Duque de Caxias, and the city of Rio de Janeiro, cities of Brazilian Southeast in the period from 1986 to 2008 did not identify the distance from patient’s residence to the clinic as a factor to impair adherence to treatment. The study suggests that if treatment is properly done, contextual factors can be minimized [24].

Therefore, it is assumed, in general, that the patient is more likely to be dropout when the residence is far from the treatment center due to travel costs and time taken. While this is true to some extent, treatment dropout rates are quite high, even for patients closer to the center. In fact, some patients prefer a place farther from the residence to remain anonymous [15].

Also in relation to housing, the study by Heukelbach and colleagues shows that people who have moved to another residence were more vulnerable to poor adherence. These people may lose their links with community health workers and other professionals from the centers of primary health care, and other factors that change in life, when moving to another place [15].

The abandonment and the irregularity of leprosy treatment has always been a matter of concern, since they may involve maintaining the chain of transmission and development of sequelae and disabilities and resistance to MDT. Whatever the causes related to noncompliance with treatment, the scientific literature suggest converge to better integration between professionals and users, valuing the psychological side and a proper motivation and education about the disease in order to obtain better treatment adherence and effective assistance to leprosy patients.

## Conclusion

It is evident that the recent scientific literature concerning the subject still presents some differences about factors that affect the dropout problem on leprosy patients treatment. It was observed that the causes responsible for non-compliance to treatment varies depending on the region that the study was conducted. However a common thread among the authors is that regardless the factor that leads to dropout treatment, there must be a more efficient health service and commitment by patient. More studies are needed in the area that approaches the particularities of each region and each patient in order to treatment happen more effectively, thus reducing the number of leprosy patients who do not adhere to the treatment properly.

## Competing interests

The authors declare that they have no competing interests.

## Authors’ contribution

RJSG, NLRS, JVP, SMFP and GPO participated in the acquisition of data and revision of the manuscript. RJSG, SMFP, LCA, VEV and FLAF conceived the study, determined the design and interpreted the data. RJSG, NLRS and JVP drafted the manuscript. All authors read and gave final approval for the version submitted for publication.
